# SMT19969 as a treatment for *Clostridium difficile* infection: an assessment of antimicrobial activity using conventional susceptibility testing and an *in vitro* gut model

**DOI:** 10.1093/jac/dku324

**Published:** 2014-09-03

**Authors:** S. D. Baines, G. S. Crowther, J. Freeman, S. Todhunter, R. Vickers, M. H. Wilcox

**Affiliations:** 1 Department of Life and Medical Sciences, University of Hertfordshire, Hatfield AL10 9AB, UK; 2 Leeds Institute for Biomedical and Clinical Sciences, University of Leeds, Leeds LS2 9JT, UK; 3 Department of Microbiology, Leeds Teaching Hospitals NHS Trust, The General Infirmary, Old Medical School, Leeds LS1 3EX, UK; 4 Summit plc, 85b Park Drive, Milton Park, Abingdon, Oxfordshire OX14 4RY, UK

**Keywords:** *Clostridium difficile*, antibiotics, cytotoxin, microflora, gut model, NAP1/027

## Abstract

**Objectives:**

We investigated the efficacy of the novel antimicrobial agent SMT19969 in treating simulated *Clostridium difficile* infection using an *in vitro* human gut model.

**Methods:**

Concentrations of the predominant cultivable members of the indigenous gut microfloras and *C. difficile* (total and spore counts) were determined by viable counting. Cytotoxin titres were determined using cell cytotoxicity and expressed as log_10_ relative units (RU). Clindamycin was used to induce simulated *C. difficile* PCR ribotype 027 infection. Once high-level cytotoxin titres (≥4 RU) were observed, SMT19969 was instilled for 7 days. Two SMT19969 dosing regimens (31.25 and 62.5 mg/L four times daily) were evaluated simultaneously in separate experiments. MICs of SMT19969 were determined against 30 genotypically distinct *C. difficile* ribotypes.

**Results:**

SMT19969 was 7- and 17-fold more active against *C. difficile* than metronidazole and vancomycin, respectively, against a panel of genotypically distinct isolates (*P* < 0.05). Both SMT19969 dosing regimens demonstrated little antimicrobial activity against indigenous gut microflora groups except clostridia. SMT19969 inhibited *C. difficile* growth and repressed *C. difficile* cytotoxin titres in the gut model.

**Conclusions:**

These data suggest that SMT19969 is a narrow-spectrum and potent antimicrobial agent against *C. difficile*. Additional studies evaluating SMT19969 in other models of *C. difficile* infection are warranted, with human studies to place these gut model observations in context.

## Introduction

*Clostridium difficile* infection (CDI) is the leading cause of infectious nosocomial diarrhoea. It is the aetiological agent of pseudomembranous colitis and is implicated in ∼30% of cases of antibiotic-associated diarrhoea.^1,2^ Most antimicrobials have been implicated, but clindamycin, third-generation cephalosporins and aminopenicillins are particularly noted for their propensity to induce CDI.^3^ Despite improved clinical management strategies for CDI, healthcare costs for treating CDI remain high and have been estimated in the USA at US$1.1–3.2 billion.^4,5^ Following the emergence of *C. difficile* PCR ribotype 027, the incidence of CDI in the UK increased markedly, but has subsequently declined. Metronidazole and vancomycin were considered for many years to be similarly effective, but later studies indicated that the former antimicrobial agent is inferior, particularly in severe CDI.^6–10^ Fidaxomicin is active *in vitro* and *in vivo* against *C. difficile*,^11,12^ and although a fidaxomicin-resistant *C. difficile* was isolated from a recurrent CDI patient (MIC of 16 mg/L) during Phase III clinical trials,^11^ the significance of this result remains to be determined. Recurrent CDI remains a therapeutic challenge, despite the recent availability of fidaxomicin, which is less efficacious against CDI due to PCR ribotype 027.^6^

We have previously described an *in vitro* human gut model of CDI that yields results consistent with *in vivo* data. The aim of this study was to evaluate the effects of the investigational drug SMT19969 on clindamycin-induced growth and toxin production in this gut model, using an epidemic *C. difficile* NAP1/027 strain.

## Materials and methods

### *C. difficile* strains

The *C. difficile* PCR ribotype 027 strain (CD 210) evaluated in the *in vitro* human gut model was isolated during an outbreak of CDI at the Maine Medical Centre (Portland, ME, USA) in 2005 and was supplied courtesy of Dr Rob Owens. A panel of clinical *C. difficile* strains were evaluated for their antimicrobial susceptibilities to metronidazole, vancomycin and SMT19969, comprising 30 genotypically distinct PCR ribotypes (PCR ribotypes: 002, 003, 005, 009, 010, 011, 014, 015, 017, 018, 019, 014/20, 023, 026, 027, 035, 044, 045, 046, 050, 056, 060, 063, 064, 067, 078, 085, 103, 106 and 153) and also an internal control strain (E4, ribotype 010) previously demonstrated to be intermediately susceptible to metronidazole.^13^

### Antimicrobial susceptibility testing

Antimicrobial susceptibilities were determined using a previously described (Wilkins Chalgren) agar incorporation method; this technique can detect reduced susceptibility of *C. difficile* to metronidazole.^14^

### *In vitro* human gut model

We have described previously the use of a triple-stage chemostat human gut model to study the interplay between antimicrobial agents, the indigenous gut microflora and *C. difficile*.^15^ The gut model was validated against physicochemical and microbiological measurements from the intestinal contents of sudden death victims.^16^ The gut model is, however, limited by its inability to simulate immunological and secretory events that occur in the human colon. The model comprises three pH-maintained (pH 5.5 ± 0.2, vessel 1; pH 6.2 ± 0.2, vessel 2; pH 6.8 ± 0.2, vessel 3) fermentation vessels, top-fed by growth medium at a controlled rate (dilution rate = 0.015 h^−1^). The gut model is inoculated with a faecal emulsion (∼10% w/v in pre-reduced PBS) prepared from *C. difficile*-negative faeces of five healthy elderly (>65 years) volunteers. Faecal donors were in good health and had received no antimicrobial therapy for at least 3 months prior to commencement of this study.

### Experimental design

**Figure 1.**
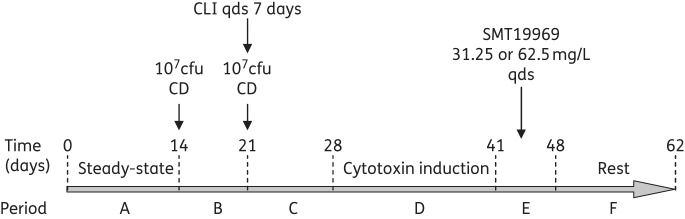
Time scheme in gut model experiments with SMT19969. CLI, clindamycin; CD, *C. difficile* spores; qds, four times daily.

Time periods for this experiment are displayed in Figure 1. SMT19969 was evaluated in separate experiments: (i) 31.25 mg/L four times daily dosing regimen for 7 days; and (ii) 62.5 mg/L four times daily for 7 days. These dosing regimens were selected to mimic 1× and 2× the clinical regimen for vancomycin in treating CDI. Following inoculation of the gut model with faecal emulsion (day 0), the media pump was started and no further interventions were made for 13 days (period A). Gut microflora were enumerated every 2 days. *C. difficile* spores (10^7^ cfu) were prepared as described previously^12^ and inoculated into vessel 1 on day 14 (period B). Viable counts of *C. difficile* and the indigenous gut microflora and *C. difficile* cytotoxin titres (Vero cell cytotoxicity assay) were monitored daily. After 7 days a further inoculum of *C. difficile* spores was instilled into vessel 1, followed by 33.9 mg/L of clindamycin (four times daily for 7 days, period C), which was instilled to reflect the concentration observed in faeces of patients and volunteers.^17^ Instillation of SMT19969 commenced once high-level cytotoxin titres ≥4 log_10_-relative units (RU) were observed on at least two consecutive days (period E). Following cessation of antimicrobial agent instillation, gut microflora populations and *C. difficile* cytotoxin titres were monitored for a further 14 days (period F).

### Enumeration of gut microflora and *C. difficile* cytotoxin titres

Gut bacterial populations and *C. difficile* concentrations were determined as described previously.^18^ Gut microflora populations cultured were total facultative anaerobes, total anaerobes (facultative + obligate), lactose-fermenting Enterobacteriaceae (LFE), enterococci, lactobacilli, bifidobacteria, total *Clostridium* spp., *Bacteroides fragilis* group (BFG), *C. difficile* total viable counts (vegetative *C. difficile* + spores) and *C. difficile* spore viable counts. *C. difficile* cytotoxin production was monitored using a Vero cell cytotoxicity assay as described previously.^18^ Indigenous gut microflora populations from vessel 1 of the gut models were not determined; only *C. difficile* total viable counts, spore counts and cytotoxin titres were quantified. Possible emergence of *C. difficile* with reduced susceptibility to SMT19969 was monitored by inoculating aliquots from the model onto Brazier's CCEY agar incorporating 0.5 mg/L (4× MIC) of SMT19969 throughout the experiment.

### Determination of SMT19969 and clindamycin concentrations

Concentrations of clindamycin achieved in each of the vessels of the gut model were determined using a microbiological bioassay with the indicator organism *Kochuria rhizophila* ATCC 9341 as described previously.^18^ Concentrations of SMT19969 achieved in each of the vessels of the gut model were determined using a microbiological bioassay with the indicator organism *Enterococcus faecalis* ATCC 29212. Samples (1 mL) for assay of antimicrobial concentrations in gut model contents were removed daily during the gut model experiments and stored at −20°C (bioassay and HPLC) and assayed retrospectively. Briefly, for bioassays performed with *E. faecalis*, the indicator organism was inoculated onto a fresh Columbia blood agar plate and incubated aerobically for 24 h at 37°C. One millilitre of a standard suspension (0.5 McFarland, 1 × 10^7^ cfu) in sterile saline was inoculated into 100 mL molten Mueller–Hinton agar and mixed gently by inversion. Bioassay dishes were incubated in air at 37°C overnight, and zone inhibition diameters measured with callipers.

#### Statistical analysis of susceptibility data

MIC data were log_2_-transformed and assessed for normality and homogeneity of variance using Minitab version 16 and SPSS version 20. Non-parametric testing (Kruskal–Wallis and *post hoc* tests) for significance was performed using these statistical analysis packages. *P* < 0.05 was considered to be statistically significant.

## Results

### Antimicrobial susceptibility testing

SMT19969 was 7- and 17-fold more active than metronidazole and vancomycin, respectively (*P* < 0.05), against the panel of 30 genotypically distinct *C. difficile* PCR ribotypes; metronidazole was twice as active as vancomycin (*P* < 0.05) (Table 1).

**Table 1.** Antibiotic susceptibilities (mg/L) of *C. difficile* to metronidazole (MTZ), vancomycin (VAN) and SMT19969

	MTZ	VAN	SMT19969
Geometric mean MIC	0.70	1.53	0.090
MIC_50_	0.50	2.00	0.125
MIC_90_	2.00	2.00	0.125

### *In vitro* gut model experiments

#### Observations during equilibration period

**Figure 2.**
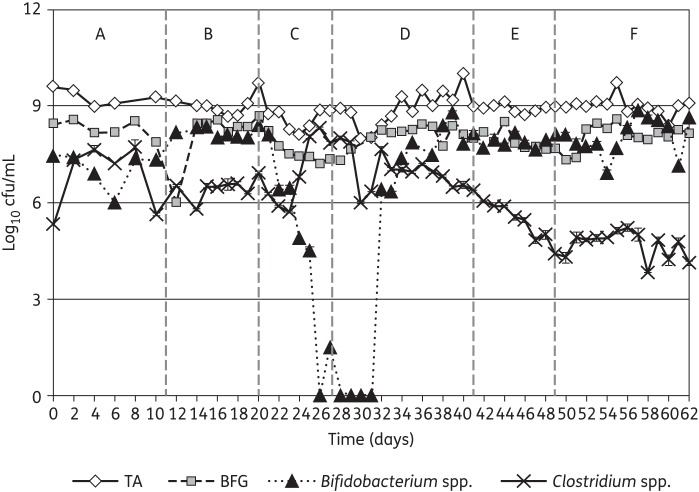
Mean viable counts (±SE) of obligate anaerobe bacterial groups (log_10_ cfu/mL) in vessel 3 of the SMT19969 250 mg/L/day gut model (period E). TA, total anaerobes (obligate and facultative).

**Figure 3.**
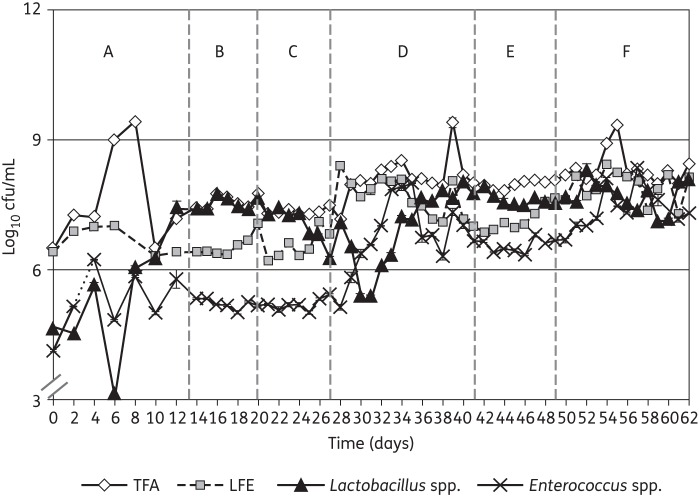
Mean viable counts (±SE) of facultative anaerobe bacterial groups (log_10_ cfu/mL) in vessel 3 of the SMT19969 250 mg/L/day gut model (period E). TFA, total facultative anaerobes.

The results observed in vessel 3 of the gut model are shown given their close similarity to data for vessel 2. Additionally, only the gut microflora viable counts from the highest dosage (250 mg/L/day SMT19969) gut model are depicted in this study due to the close similarity between the two experiments. Viable counts of all bacterial groups increased during the steady-state period compared with those observed on the first day of the experiment (Figures 2 and 3). Gut microfloras in vessels 2 and 3 of the gut models were dominated by obligate anaerobes, particularly *Bifidobacterium* spp. and BFG, with lower observed viable counts of facultatively anaerobic bacterial groups. Viable counts of indigenous gut microfloras were largely stable by the end of this period.

#### Observations following instillation of C. difficile spores

**Figure 4.**
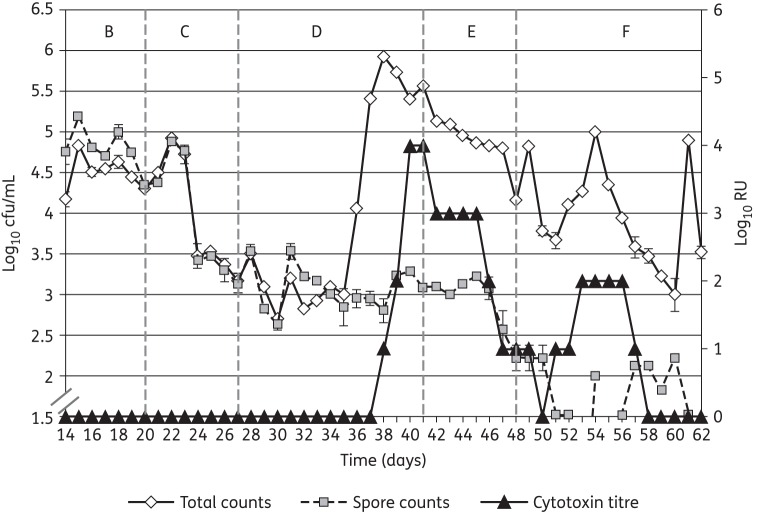
*C. difficile* total viable counts, spore counts (log_10_ cfu/mL) and cytotoxin titres (RU) in vessel 3 of the gut model dosed with 250 mg/L/day SMT19969 (period E).

**Figure 5.**
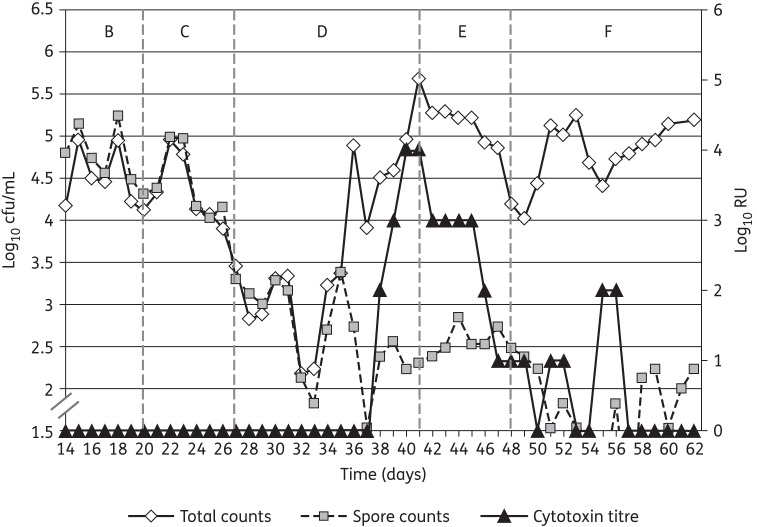
*C. difficile* total viable counts, spore counts (log_10_ cfu/mL) and cytotoxin titres (RU) in vessel 3 of the gut model dosed with 125 mg/L/day SMT19969 (period E).

Single inocula of *C. difficile* spores (2 × 10^7^ cfu) were instilled into the gut models on day 14 of each experiment. Although some minor fluctuations in viable counts of the indigenous gut microflora were observed following instillation of *C. difficile* spores, no consistent trend was seen across both gut models. *C. difficile* viable counts in vessel 1 of both gut models declined at a similar rate after the initial inoculation of *C. difficile* spores and there was no evidence of spore germination, proliferation or cytotoxin production (Figures 4 and 5). *C. difficile* viable counts in vessels 2 and 3 of the gut models increased during the initial stages of the period, as a consequence of washout from vessel 1, then declined for the remainder of the period. There was no evidence of *C. difficile* germination, proliferation or cytotoxin production (Figures 4 and 5).

#### Observations following instillation of clindamycin

Instillation of clindamycin facilitated similar alterations in viable counts of gut microfloras in both gut models. A sustained reduction in viable counts of lactobacilli was observed following clindamycin instillation (1.5–2.0 log_10_ cfu/mL), while transient minor declines in viable counts of some other groups of obligate anaerobes was observed (Figures 3 and 4). Marked declines in viable counts of bifidobacteria were observed during clindamycin instillation (6.0–7.0 log_10_ cfu/mL) in both gut models. Additionally, BFG viable counts declined by 1.0 log_10_ cfu/mL (Figure 2). Conversely, viable counts of *Enterococcus* spp. increased markedly following cessation of clindamycin instillation (Figure 3). *C. difficile* remained quiescent during clindamycin instillation in both gut models; populations comprised principally spores, and cytotoxin was undetectable (Figures 4 and 5). Bacterial groups deleteriously affected by clindamycin instillation largely recovered by the end of the cytotoxin induction period. LFE viable counts increased markedly during the cytotoxin induction period.

*C. difficile* germination and proliferation were observed 8 days (vessel 2, day 35) and 9 days (vessel 3, day 36) after cessation of clindamycin instillation (Figures 4 and 5). Cytotoxin was demonstrated in vessel 1 of both gut models 11 days after cessation of clindamycin instillation. Cytotoxin titres increased to 4 RU in vessels 2 and 3 of both gut models, but remained at 2 RU in vessel 1. Instillation of SMT19969 commenced 15 days after cessation of clindamycin instillation (day 42) in both gut models.

### Clindamycin concentrations detected in the gut models

Mean clindamycin concentrations (mg/L) observed during the instillation period in the 125 and 250 mg/L/day gut models were 48.4 and 47.7 (vessel 1), 35.2 and 39.9 (vessel 2), and 36.2 and 38.8 (vessel 3), respectively. Clindamycin was quickly washed out of the gut model vessels following cessation of instillation, and was undetectable 6 days after cessation in all vessels of both experiments.

### Effect of SMT19969 on the indigenous gut microflora and *C. difficile*

Both SMT19969 dosing regimens demonstrated little antimicrobial activity against the indigenous gut microflora in the two gut models (Table 2). Total *Clostridium* spp. was the only indigenous gut microflora group for which viable counts declined following SMT19969 instillation (Figure 2). Modest increases in viable counts of LFE were observed following instillation of both dosing regimens. Vegetative forms of *C. difficile* (SMT19969 MIC = 0.125 mg/L) declined during the SMT19969 instillation period (∼1.5 log_10_ cfu/mL over 7 days) in all vessels of both gut models (Figures 4 and 5). Following cessation of SMT19969 administration, continued declines in *C. difficile* counts by up to a further 2 log_10_ cfu/mL were observed in the 250 mg/L/day model (Figure 4). *C. difficile* cytotoxin titres declined rapidly following commencement of SMT19969 instillation, with overall declines of 2, 4 and 3 RU observed in vessels 1, 2 and 3, respectively, from days 42 to 48. Following cessation of SMT19969 instillation, *C. difficile* vegetative populations remained detectable and increased slightly by the end of the experiment. Cytotoxin was undetectable for the final 6 and 5 days of the 125 and 250 mg/L/day gut models, respectively.

**Table 2.** Overall change in viable counts (log_10_ cfu/mL) of gut microflora groups and *C. difficile*, and cytotoxin titres (CYT, RU) during SMT19969 dosing (days 42–48)

Bacterial group	125 mg/L/day		250 mg/L/day	
	vessel 2	vessel 3	vessel 2	vessel 3
TFA	NC	NC	NC	NC
LFE	+1.0	+0.6	+0.6	+0.6
BFG	NC	NC	NC	NC
Bifidobacteria	NC	NC	NC	NC
Lactobacilli	NC	NC	NC	NC
Clostridia	−2.0	−1.7	−2.0	−1.4
Enterococci	NC	NC	NC	NC
Total anaerobes	NC	NC	NC	NC
CD TVC	−1.5	−1.5	−1.7	−1.4
CD SP	−1.3	NC	−1.1	−0.9
CD CYT	−4.0	−3.0	−4.0	−3.0

TFA, total facultative anaerobes; TVC, total viable count; SP, spore count; CD, *C. difficile*; NC, no substantial change (±0.5 log_10_ cfu/mL).

### Isolation of *C. difficile* on breakpoint agars

No *C. difficile* were isolated from SMT19969 breakpoint agar (4× MIC) plates during the course of the experiments.

## Discussion

The efficacy of existing CDI therapies can be affected by: (i) poor drug pharmacokinetics, e.g. very low concentrations of metronidazole within the colonic lumen; (ii) fear of bacterial resistance emergence (e.g. VRE following vancomycin exposure), although a recent study suggested that these fears may be unfounded;^19^ and (iii) reduced efficacy against hypervirulent *C. difficile* ribotypes.^6^ Emergence of resistance or reduced susceptibility is a concern for metronidazole,^14^ especially given its aforementioned poor bioavailability, although currently no link between reduced susceptibility and treatment failure has been established.^20^ Emergence of resistance to fidaxomicin has been documented in a single instance in the clinical setting; however, rigorous serial passage studies and human gut model studies have failed to document similar findings.^11^ Similarly, serial passage studies with SMT19969 demonstrated a low propensity of resistance development with mutation frequencies <3.09 × 10^−9^.^21^

The results of the present study show that SMT19969, a novel, narrow-spectrum, non-absorbable antimicrobial agent, has high potency against *C. difficile* in standard agar incorporation MIC experiments. SMT19969 MICs were 7- and 17-fold lower than corresponding metronidazole and vancomycin MICs and are amongst the most potent anti-*C. difficile* antimicrobial agents reported. The narrow spectrum of antibacterial activity demonstrated from SMT19969 susceptibility testing studies^22^ was reinforced in the present gut model experiments, in that the only indigenous microflora group adversely affected following SMT19969 instillation was *Clostridium* spp. (2 log_10_ cfu/mL decline). This observed anticlostridial activity reflects prior *in vitro* susceptibility studies for this genus,^23^ particularly *Clostridium innocuum*, which is often found within the gut models in high concentrations.

The gut model has proven a valuable tool in the drug development setting, both to assess the likelihood of CDI induction by antibiotics, and to determine the efficacies of novel antimicrobial agents for treating CDI. Indeed, clinical observations have reflected *in vitro* observations in predicting suboptimal efficacy of the toxin-binding polymer tolevamer,^9,24^ the superiority of fidaxomicin compared with vancomycin in treating recurrent CDI and the low propensity of certain clinical antimicrobial agents to induce CDI.^25–27^ Prior gut model studies showed that metronidazole and vancomycin adversely affect the anaerobic gut microflora (BFG and/or bifidobacteria) and shift the balance from anaerobe- to facultative-anaerobe-dominated microflora (LFE and/or lactobacilli and/or enterococci), which is a similar microflora shift to that observed for antimicrobial agents with a recognized propensity to induce CDI (clindamycin, ceftriaxone and cefotaxime). In the present study, neither SMT19969 regimen adversely affected the normal, anaerobe-dominated microflora in the gut model vessels, which are analogous to the distal end of the colon, which may confer this antimicrobial an advantage over existing therapies. Fidaxomicin was recently demonstrated to inhibit *Bifidobacterium* spp. in three of four gut model experiments and facilitated a microflora dominated by LFE, with reduced BFG concentrations.^12^ Despite these microflora changes, fidaxomicin instillation was not associated with recurrent simulated CDI in the gut model; it was suggested that the persistence of supra-MIC of fidaxomicin prevents *C. difficile* spore germination and subsequent simulated recurrence. Metronidazole was demonstrated as an ineffective treatment for simulated CDI in the gut model in prior studies, where poor bioactive concentrations of drug within the gut model and signs of recurrent simulated CDI were observed.^12^

Instillation of SMT19969 into both gut models elicited an immediate decline in cytotoxin production. Cytotoxin titres declined substantially by the end of period E, and were at the limit of detection 2 days later in both high and low SMT19969 dosage models. The decline in *C. difficile* total viable counts observed following SMT19969 instillation was incremental over several days and indicated a bacteriostatic mechanism of action for this antibiotic under these experimental conditions. However, in standard *in vitro* testing SMT19969 demonstrates bactericidal activity with >5 log reduction in cfu/mL at 24 h.^28^ These declines in *C. difficile* concentrations following SMT19969 instillation in the gut model are similar to, or better than, declines observed following instillation of vancomycin into the gut model,^29,30^ although in the present study total viable counts remained above spore counts in the SMT19969 dosing period. In a recent *in vivo* study using the hamster model of CDI, SMT19969 elicited complete clearance of viable *C. difficile* following commencement of dosing (R. Vickers, unpublished results), and in the Phase I clinical trial of SMT19969, clostridial viable counts were below the lower limits of detection for viable counting (whereas other gut microflora groups were unaffected).^31^ The differences between the present *in vitro* study and recent *in vivo* studies with SMT19969 probably reflect a suboptimal formulation used in the present experiments that has been shown to result in significantly reduced efficacy in the hamster model of CDI (R. Vickers, unpublished results). Visible precipitates of SMT19969 were observed following instillation of the drug and persisted for the duration of the experiment in both gut models. Poor solubility also hampered efforts to determine the dissolved (bioavailable) fraction of SMT19969, and therefore some caution must be exercised when interpreting the antimicrobial activity of SMT19969 in these gut model studies. Cytotoxin titres increased slightly during period F (rest period), but to a relatively low titre (2 RU), and titres were below the limits of detection for the last 5–6 days of both experiments. The sporadic spikes in total viable count and low-level cytotoxin observed during period F may be due to the release of vegetative *C. difficile* from biofilms within the gut models; both the organism and its cytotoxins have been demonstrated to be present in the complex, multispecies biofilms within the gut model vessels (G. S. Crowther, M. H. Wilcox and J. Freeman, unpublished results).^32^ Instillation of SMT19969 curtailed the production of cytotoxin by *C. difficile* ribotype 027, noting that prior gut model studies have consistently demonstrated a substantially longer (>23 days), but not higher, peak cytotoxin titre by this virulent ribotype.^18^

The results of the present study, combined with a good Phase I safety and tolerability profile, and superior efficacy to vancomycin in treating CDI in the hamster model,^21^ indicate that further studies with SMT19969 are warranted to determine its efficacy as a potential CDI therapeutic option. Improvements to the solubility of SMT19969, or reduced dosing to aid dissolution, may facilitate the demonstration of enhanced efficacy *in vitro* and *in vivo* in future studies. In particular, an assessment of the efficacy of SMT19969 in preventing recurrent CDI both *in vitro* and *in vivo* would help to determine whether this is an area in which this novel antimicrobial agent may outperform other CDI therapies. Indeed, the extremely narrow spectrum of antibacterial activity of SMT19969 is attractive, and theoretically suggests a reduced likelihood of CDI recurrence due to reduced perturbation of colonic colonization resistance.

## Funding

This study was initiated and financially supported by Summit plc through a Seeding Drug Discovery Award from the Wellcome Trust (grant number 091055).

## Transparency declarations

In the past two years, G. S. C. has received support to attend meetings from Novacta. M. H. W. has received research funding from Actelion, Astellas, BioMerieux, Cubist, Pfizer, Summit Plc. and The Medicines Company and consultancies and/or lecture honoraria from Actelion, Astellas, Astra-Zeneca, Bayer, Cubist, Durata, J&J, Merck, Nabriva, Novacta, Novartis, Optimer, Pfizer, Sanofi-Pasteur, The Medicines Company, VH Squared and Viropharma. All other authors: none to declare.
